# Integrated pan-cancer analysis and experimental validation reveal that syndecan-1 drives glioblastoma pathogenesis and associates with immune infiltration

**DOI:** 10.1007/s12672-026-05172-0

**Published:** 2026-05-07

**Authors:** Qing Wang, Wenqian Ai, Sisi Yu, Yi Xu, Kunjian Lei, Shigang Lv

**Affiliations:** 1 Department of Neurosurgery, The Second Affiliated Hospital, Jiangxi Medical College, Nanchang University, Nanchang, 330006 Jiangxi People’s Republic of China; 2 School of Public Health, Nanchang University, Nanchang, 330031 Jiangxi People’s Republic of China; 3 Changsha KingMed Center for Clinical Laboratory Co. Ltd., Changsha, Hunan China

**Keywords:** SDC1, Pan-cancer analysis, GBM, Prognosis, Immune infiltration, Biomarker

## Abstract

**Backgrounds:**

Syndecan-1 (SDC1) is a key transmembrane proteoglycan involved in cell adhesion and epithelial integrity. Although evidence links SDC1 to tumor progression, its pan-cancer functions and mechanistic roles are not fully understood.

**Methods:**

We defined the multidimensional role of SDC1 across cancers by leveraging multi-platform genomic and clinical data. Our study characterized its associations with patient survival, molecular mechanisms, immune contexture, and therapeutic sensitivity. Initial observations in glioma subtypessuggested a pro-tumorigenic function. Consequently, this functional role in glioblastoma (GBM) was empirically validated through a combination of cellular-level assays and animal xenograft models.

**Results:**

SDC1 expression was upregulated in numerous cancer types, correlating with poorer survival rates among patients. Receiver operating characteristic (ROC) analyses confirmed its robust diagnostic value across malignancies. Notably, a notable correlation was identified between SDC1 levels and key components within the tumor microenvironment, including CD4+/CD8 + T lymphocytes, cancer-associated fibroblasts (CAFs), and myeloid-derived suppressor cells (MDSCs). Functional assays revealed that SDC1 depletion markedly attenuated GBM cell proliferation and migration. Subsequently, the capacity for migration and tissue invasion was assessed using both experimental and animal model systems.

**Conclusion:**

Integrated bioinformatic and experimental analyses identify SDC1 as a pan-cancer biomarker with particular clinical significance in GBM. Its expression correlates with immune infiltration patterns, positioning SDC1 as a promising diagnostic and immunotherapeutic target.

**Supplementary Information:**

The online version contains supplementary material available at 10.1007/s12672-026-05172-0.

## Introduction

The global burden of malignant diseases continues to escalate, presenting substantial challenges to healthcare systems worldwide. According to 2024 epidemiological data, China reported approximately 4,909,585 new cancer diagnoses and 2,593,882 cancer-associated deaths, while the United States documented 2,491,868 incident cases and 633,864 mortalities [1]. This progressive increase in cancer incidence and mortality underscores the persistent significance of neoplasms as a public health concern [2]. Current therapeutic modalities—encompassing surgical procedures, chemotherapeutic regimens, radiation therapy, molecular-targeting approaches, and immunotherapeutic strategies—continue to yield inadequate long-term survival outcomes. Therefore, advancing more efficient methods for cancer prevention, early detection, and clinical management has become an imperative medical requirement.

As prominent transmembrane heparan sulfate proteoglycans, syndecan family members localize to cell membranes and engage in multiple biological functions, serving as essential regulators of signal transduction, cellular architecture, immune regulation, and inflammatory processes [3]. SDC1 is the most thoroughly investigated family member, characterized by a tripartite structure comprising extracellular, transmembrane, and cytoplasmic domains [4]. The extracellular segment bears heparan sulfate modifications that enable interactions with diverse ligands such as FGF, VEGF, and HGF. By facilitating ternary complex assembly involving heparan sulfate chains, ligand molecules, and high-affinity receptors, SDC1 potently augments growth factor signal transduction [5]. Beyond these functions, SDC1 acts as a critical coreceptor in Wnt signaling pathways, coordinating with Frizzled receptors to stabilize β-catenin and promote its nuclear accumulation, thereby initiating transcriptional activation that fosters tumor initiation and stem cell preservation [6].

Physiologically, SDC1 is primarily expressed in plasma cells and epithelial tissues, where it contributes to epithelial homeostasis, cellular polarization, adhesive interactions, and differentiation programs. Neoplastic transformation induces significant alterations in SDC1 expression profiles, spatial distribution, and molecular species—encompassing membrane-anchored, solubilized, and nuclear forms—converting it into a potent mediator of oncogenic progression, invasive behavior, metastatic spread, angiogenic induction, and therapeutic resistance. Such modifications consistently correlate with compromised patient survival [7]. Accumulated evidence delineates SDC1’s pleiotropic functions in tumor pathogenesis. For instance, SDC1 binding to Wnt and HGF ligands reinforces survival signaling cascades, thereby accelerating proliferation in plasma cell dyscrasias [8, 9]. It furthermore augments macrophnocytic nutrient uptake, promoting growth in breast and pancreatic malignancies [10, 11]. Within GBM contexts, SDC1 mediates TGM2 trafficking and assembles an SDC1-TGM2-FLOT1-BHMT supercomplex that enhances autophagosomal-lysosomal fusion, ultimately conferring radiotherapeutic resistance [12, 13].

Notwithstanding these advances, SDC1’s pathogenetic roles across diverse cancer lineages remain incompletely characterized. Our investigation implements consolidated data resources to evaluate SDC1’s transcriptional landscape, prognostic implications, diagnostic applicability, and functional pathway associations via Gene Set Enrichment Analysis (GSEA). We additionally delineate its connections with immunophenotypic categories, molecular classifications, infiltrating immune constituents, and chemotherapeutic sensitivity. By formulating and experimentally validating a mechanistic model of SDC1 function in GBM pathogenesis, we substantiate its oncogenic potential. This expansive multi-cancer profiling yields valuable biological insights that support SDC1’s biomarker applicability for prognostic stratification, immune microenvironment assessment, and immunotherapeutic innovation.

## Materials and methods

### Data sources

We systematically investigated SDC1 expression and genomic characteristics using multiple bioinformatics resources. Comprehensive gene expression and clinical annotation datasets spanning various human malignancies were sourced from the UCSC Xena platform (https://xena.ucsc.edu/). This integrated bioinformatics portal consolidates and harmonizes large-scale genomic data from the Genotype-Tissue Expression (GTEx) and The Cancer Genome Atlas (TCGA) projects, enabling cross-study analyses It provides pre-processed TCGA and GTEx data with normalization and batch-effect correction already applied [14]. A comparative study of SDC1 protein expression levels in normal versus tumor tissues was conducted using UALCAN (http://ualcan.path.uab.edu/analysis.html), leveraging the Clinical Proteomic Tumor Analysis Consortium (CPTAC) [15]. GEPIA3 (https://gepia3.bioinfoliu.com) facilitated examination of differential SDC1 expression patterns in GBM [16]. Genomic alteration profiles of SDC1, including mutation frequency and variant classification across tumor types, were interrogated using cBioPortal (https://www.cbioportal.org/) [17]. Based on data from the Human Protein Atlas (HPA) database (https://www.proteinatlas.org), we analyzed the subcellular localization of SDC1 [18]. Furthermore, a high-confidence interaction network (score ≥ 0.700) was generated via the STRING database (https://cn.string-db.org/), identifying 50 proteins with potential associations to SDC1 [19]. Subsequent network visualization and modular examination were performed using Cytoscape software (v3.10.1). Abbreviations for all tumors, the immune subtypes, and other terms have been added to the Abbreviations section.

### Pan-cancer diagnostic potential of SDC1

The diagnostic utility of SDC1 for cancer detection was assessed through ROC analysis. This conventional analytical technique quantifies the biomarker’s capacity to distinguish between diseased and non-diseased states across varying thresholds, using merged transcriptomic datasets from TCGA and GTEx. The R package “pROC” was employed to computationally generate ROC curves. This analysis produced area under the curve (AUC) estimates ranging between 0.5 and 1.0. According to conventional diagnostic benchmarks, AUC scores between 0.5 and 0.7 indicate low discrimination, 0.7–0.9 moderate discrimination, and scores above 0.9 indicate high discrimination.

### Prognostic analysis of SDC1 across cancers

To assess the prognostic significance of SDC1, transcriptomic profiles and corresponding clinical characteristic information for 33 malignancies were collected from TCGA through the UCSC Xena interface (https://xena.ucsc.edu/) [14]. Patient survival was evaluated by analyzing multiple clinical endpoints, including overall survival (OS), progression-free interval (PFI), disease-specific survival (DSS), and recurrence-free survival (RFS). Associations between SDC1 expression levels and clinical outcomes were evaluated through univariate Cox proportional hazards models and Kaplan-Meier survival analyses. The “survival” package in R was utilized to conduct all statistical computations, encompassing the derivation of log-rank p-values, hazard ratios (HR), and their 95% confidence intervals (CI). Final results are displayed as an integrated heatmap, with OS associations further detailed in forest plots generated with the “forestplot” package.

### GSEA for SDC1

For each cancer type in the TCGA cohort, samples were dichotomized into SDC1-high and SDC1-low groups using the median expression cutoff. The differentially expressed genes (DEGs) identified from this comparison were then subjected to GSEA with hallmark gene sets from the Molecular Signatures Database (MSigDB) (https://www.gsea-msigdb.org/gsea/index.jsp). The “h.all.v7.4.symbols.gmt” collection, comprising 50 well-defined biological pathway sets, served as our reference database. Input genes for GSEA were ranked according to log fold change (logFC) values, which reflect transcriptional differences between SDC1 expression subgroups. Using the “clusterProfiler” R package, we computed Normalized Enrichment Scores (NES) to evaluate enrichment strength and implemented False Discovery Rate (FDR) adjustment to control for multiple testing [20]. Result visualization was achieved using bubble plots generated with the “ggplot2” package, where marker characteristics enable intuitive interpretation of enrichment patterns.

### SDC1 levels are associated with tumor immune and molecular subtypes in pan-cancer

TISIDB (cis.hku.hk) integrates multi-omics data from diverse sources—including published literature, high-throughput screens, immunotherapy trials, and TCGA—to enable systematic exploration of tumor-immune crosstalk and accelerate the discovery of immunotherapy targets and biomarkers [21]. Using this repository, we examined SDC1 expression correlations with various immune-based and molecular subtype classifications across human malignancies.

### Immune infiltration analysis of SDC1

TIMER2.0 database (https://compbio.cn/timer/2) was utilized to retrieve information on immune cell infiltration associated with SDC1 expression [22]. We applied Spearman’s rank correlation analysis to examine connections between SDC1 mRNA expression and infiltration densities of 21 distinct immune cell populations across various malignancies. The present study analyzed a diverse panel of cellular constituents within the tumor microenvironment. These encompassed adaptive immune cells such as CD4 + T lymphocytes, follicular helper T cells, natural killer T (NKT) cells, γδ T cells, regulatory T cells (Tregs), B lymphocytes, and CD8 + T cells. Innate immune populations included natural killer (NK) cells, neutrophils, monocytes, macrophages, dendritic cells, eosinophils, and mast cells. Additionally, the analysis incorporated MDSCs and progenitor and precursor cells, including hematopoietic stem cells (HSCs), lymphoid progenitors, and myeloid precursors. Stromal components, namely CAFs and endothelial cells, were also evaluated. The comprehensive profiling covered cells from the monocyte lineage to provide a holistic view of the cellular landscape.

### Drug sensitivity analysis for SDC1

We systematically examined SDC1-associated drug responses across malignancies by merging drug response information from the GDSC and CTRP databases with SDC1 gene expression data. Using the Gene Set Variation Analysis (GSVA) platform (https://guolab.wchscu.cn/GSCA/#/) [23], we identified multiple pharmacological agents with statistically significant correlations (FDR < 0.05) with SDC1 expression levels. These findings advance our understanding of SDC1’s therapeutic implications and inform potential targeted treatment strategies.

### Clinical correlation and prognostic role of SDC1 in glioma patients

We evaluated SDC1’s clinical relevance in glioma by analyzing its expression patterns across GBM, lower-grade glioma (LGG), and integrated glioma cohorts in relation to essential clinical variables: patient gender and age, key molecular markers (IDH mutation and 1p/19q co-deletion status), and histopathological classification based on WHO criteria. Subsequent analyses focused on whether high SDC1 levels correlated with poorer overall patient survival. Using the R package ‘maxstat’, we established an optimal expression cutoff to categorize patients into elevated and reduced expression levels. We performed survival analysis to correlate SDC1 expression levels with patientOS. This involved generating survival curves via the `survfit` function from the `survival` package in R and comparing them with the log-rank test. To determine its independent prognostic value, we subjected SDC1 expression data from the glioma cohort to both univariate and multivariate Cox proportional-hazards regression analyses.

### Single-sample gene set enrichment analysis (ssGSEA) analysis in glioma patients for SDC1

ssGSEA uses RNA-seq transcriptomic profiles to derive quantitative scores for immune cell infiltration, a methodology commonly employed in research on solid malignancies. We implemented this method to evaluate 29 immune cell types and functional signatures in glioma specimens obtained from TCGA. Cohort stratification was performed based on the median SDC1 expression, dividing samples into elevated- and reduced-expression subtypes. Additionally, the ‘ESTIMATE’ algorithm implemented in R was utilized to calculate tumor purity and generate corresponding estimate, immune, and stromal scores for each glioma sample.

### Cell culture

The human GBM cell lines U87 and U251 were obtained from the American Type Culture Collection (ATCC). Cells were cultured in a humidified incubator at 37 °C with 5% CO₂, using Dulbecco’s Modified Eagle Medium (DMEM) supplemented with 10% fetal bovine serum (FBS; Gibco) and 1% penicillin-streptomycin.

### Western blot analysis and quantitative real-time PCR (qRT-PCR)

Total protein lysates were prepared using RIPA buffer and quantified by the BCA assay prior to downstream molecular profiling. For protein separation, lysates containing 20 µg of protein per lane were electrophoresed on 10% SDS-polyacrylamide gels and then electrotransferred to PVDF membranes. Following blocking with 10% BSA, the membranes were probed with specific primary antibodies at 4 °C for 16 h. Following washes, the membranes were incubated with horseradish peroxidase (HRP)-conjugated secondary antibodies for 1 h at room temperature. Signals were visualized by ECL, and their intensities were quantified using ImageJ software. For gene expression assessment, total RNA was extracted with TRIzol reagent and subsequently reverse-transcribed. Subsequently, qPCR was performed using SYBR Green. The following primer sequences were employed: SDC1-F: 5′-CTGCCGCAAATTGTGGCTAC-3′,

SDC1-R: 5′-TGAGCCGGAGAAGTTGTCAGA-3′, GAPDH-F: 5′-GGAGCGAGATCCCTCCAAAAT-3′, GAPDH-R: 5′-GGCTGTTGTCATACTTCTCATGG-3′.

### CCK-8 assay

Cell proliferation following SDC1 knockdown was evaluated with the CCK8, obtained from Solarbio in China. U87 and U251 cells were seeded into 96-well plates at a density of 2 × 10³ cells per well. At 0, 24, 48, 72, 96, and 120-hour intervals, each well received an addition of 10 µL of CCK-8 solution. Following a 60-minute incubation period, cellular viability was quantified by detecting absorbance at 450 nm using a microplate reader.

### Colony formation assay

For the colony formation assay, U87 and U251 cells were plated in six-well plates (100 cells/well) and incubated at 37 °C for 14 days. Following incubation, colonies were fixed with 4% paraformaldehyde and visualized by staining with 0.5% crystal violet. Following a PBS wash, the number of colonies was quantified.

### EdU assay

We assessed proliferative activity by performing 5-ethynyl-2′-deoxyuridine (EdU) incorporation analysis. Cells planted in six-well plates were incubated with 10 µM EdU for 2–4 h to label nascent DNA. To prepare for subsequent staining, specimens were first fixed in 4% paraformaldehyde and then permeabilized by incubation with 0.3% Triton X-100 for 10 min, and subsequently stained with Hoechst dye for nuclear visualization for 5 min. Imaging was conducted using fluorescence microscopy to quantify proliferating cells.

### Transwell assay (without Matrigel for migration)

Cell migration capacity was evaluated using Transwell systems. Human glioblastoma cell lines U87 and U251 were suspended in medium without serum and placed in the upper chambers. After 24 h, cells that had traversed the membrane were fixed with 4% paraformaldehyde and subjected to staining with 0.1% crystal violet solution. Migratory cell numbers were quantified by averaging counts from five randomly selected microscopic fields per specimen.

### Scratch wound assay

The migratory ability of U87 and U251 cells was assessed by first culturing them in 6-well plates to reach full confluence. Uniform linear scratches were then introduced into the cell monolayers using a sterile 10 µL pipette tip. Photographic documentation was obtained at 0 h post-wounding and again after 36 h of incubation to monitor wound-healing dynamics.

### Nude mouse xenograft tumor model

Five-week-old male BALB/c nude mice (GemPharmatech, China) were used to develop orthotopic glioma xenograft models. A total of 1.5 × 10⁵ U251-shVector or U251-shSDC1 cells were injected intracranially. Tumor progression was tracked via bioluminescence imaging, and survival was documented. Mice were euthanized humanely at study endpoints.

### Statistical analysis

Patients were stratified into high and low SDC1 expression groups, and their survival outcomes were compared using Kaplan-Meier analysis with a two-sided log-rank test. The predictive accuracy of SDC1 expression was assessed via time-dependent ROC analysis, with the AUC calculated using the R package “pROC”. Furthermore, univariate and multivariate Cox proportional hazards regression models were employed to evaluate the independent prognostic value of SDC1 across all cohorts. Statistical significance was set at a two-sided p-value < 0.05. In in vitro experiments, group comparisons were analyzed by Student’s t-test. All p-values derived from the pan-cancer analysis were adjusted for multiple testing using the FDR approach. All analyses were performed with SPSS Statistics (version 25) and R (version 4.4.2).

## Results

### Basic information of SDC1

**Fig. 1:**
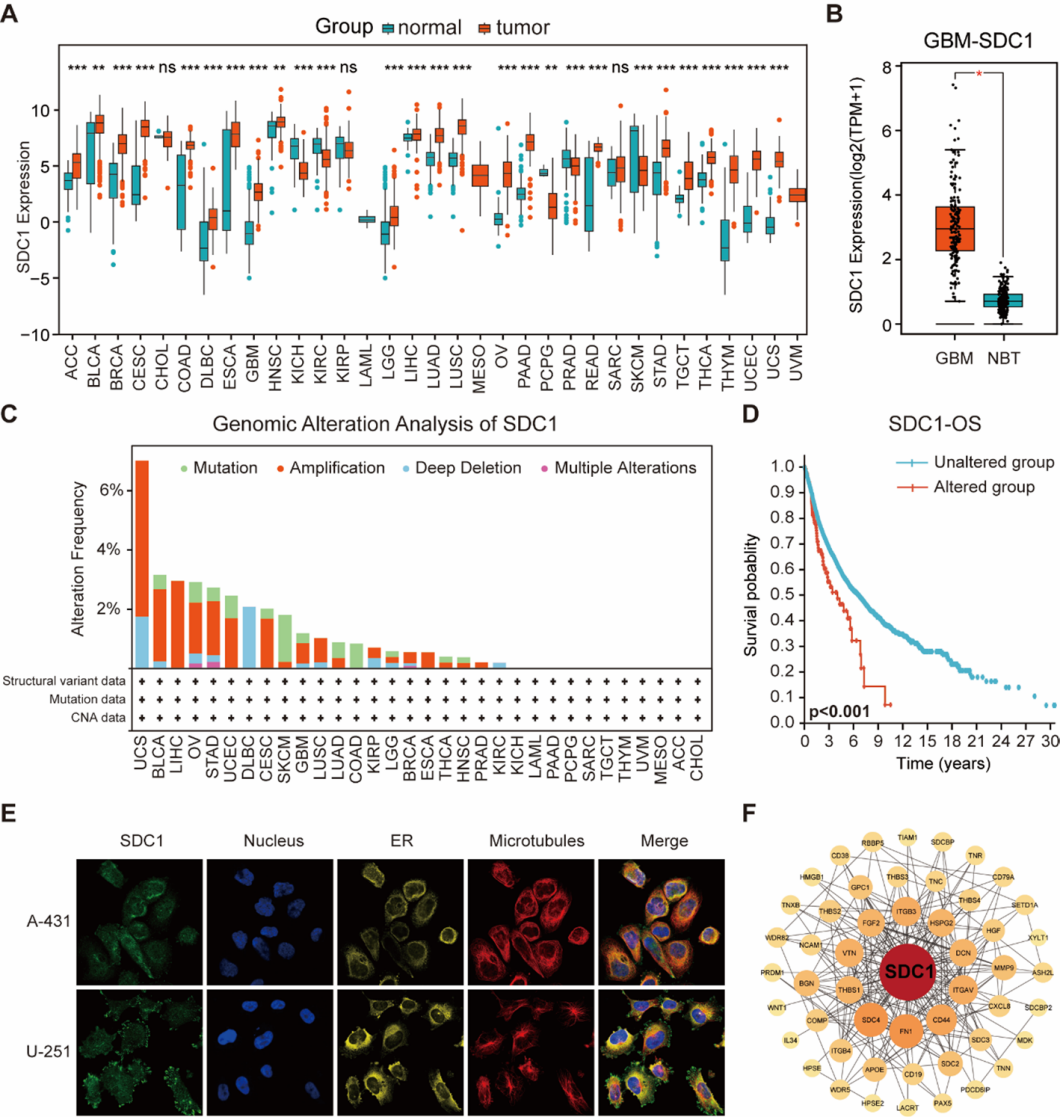
Basic Information of SDC1. **A** Comparison of SDC1 transcriptional profiles between tumor and normal tissues was performed utilizing combined data from TCGA and GTEx. Red markers and boxes indicate tumor samples, whereas regular counterparts are shown in blue. **B** Comparison of SDC1 expression in GBM versus non-neoplastic brain tissue. **C** Bar plot showing SDC1 mutations across 32 cancer types based on TCGA Pan-Cancer Atlas studies. **D** OS analysis based on Kaplan-Meier curves, comparing patients with and without SDC1 mutations. **E** Immunofluorescence analysis of SDC1 spatial distribution in A-431 and U-251 cells using the HPA database. **F** Top 50 SDC1-associated proteins. Asterisks denote statistical significance levels: ns (not significant, *P* > 0.05), * (*P* < 0.05), ** (*P* < 0.01), and *** (*P* < 0.001)

SDC1 mRNA levels were profiled by integrating data from TCGA and GTEx databases. Comparative analysis across 27 human cancers identified significant expression variations: SDC1 was elevated in 22 tumor types and reduced in 5 others (Fig. 1A). These pan-cancer observations were validated in GBM through GEPIA3 analysis (Fig. 1B). At the protein level, UALCAN data revealed increased SDC1 expression in Breast invasive carcinoma (BRCA), Uterine Corpus Endometrial Carcinoma (UCEC), lung cancer, Pancreatic adenocarcinoma (PAAD), and GBM, but decreased levels in Clear cell RCC and liver cancer (Fig. S1A).

Genomic alteration analysis using cBioPortal identified UCS as having the highest SDC1 mutation frequency (7.02%), driven mainly by amplification events—a pattern also observed in GBM (Fig. 1C). Prognostic evaluation across 33 cancer types demonstrated that patients with SDC1 mutations had significantly reduced OS compared to those without alterations (Fig. 1D), with similar patterns observed for DSS and PFI (Fig. S1B-D). Immunofluorescence imaging from HPA revealed focal adhesion localization of SDC1 in A-431 and U-251 cell models (Fig. 1E). Additionally, to elucidate the functional context of SDC1, a protein-protein interaction (PPI) network was constructed using the STRING database. This high-confidence network incorporated 50 SDC1-associated proteins, such as SDC2, SDC4, and SDCBP (Fig. 1F).

#### Diagnostic value of SDC1 in pan-cancer

**Fig. 2:**
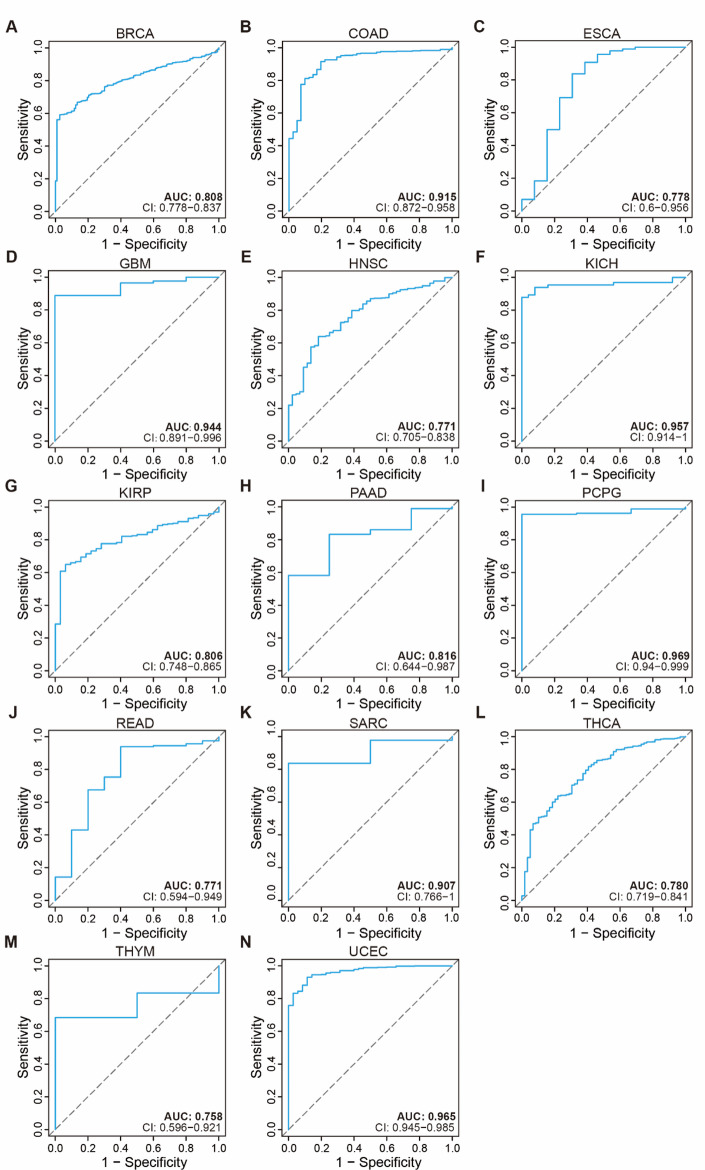
SDC1 performance in distinguishing malignant from normal tissues across various cancers. **A-N** SDC1 demonstrated moderate to strong diagnostic accuracy (AUC > 0.7) across 14 cancer types, with six cancers achieving AUC > 0.9

The diagnostic performance of SDC1 was evaluated by constructing ROC curves, which compared its expression levels in malignant versus non-malignant tissues (Fig. 2). The gene showed moderate to strong discriminatory power (AUC > 0.7) in distinguishing malignancies from normal tissues across 14 cancer types. Six cancers—Colon adenocarcinoma (COAD), GBM, Kidney Chromophobe (KICH), Pheochromocytoma and Paraganglioma (PCPG), Sarcoma (SARC), and UCEC—showed outstanding diagnostic accuracy (AUC > 0.9). Another eight tumor types also demonstrated considerable diagnostic potential, including BRCA, Esophageal carcinoma (ESCA), Head and Neck squamous cell carcinoma (HNSC), Kidney renal papillary cell carcinoma (KIRP), PAAD, Rectum adenocarcinoma (READ), Thyroid carcinoma (THCA), and Thymoma (THYM).

### Prognostic role of SDC1 in cancers

**Fig. 3:**
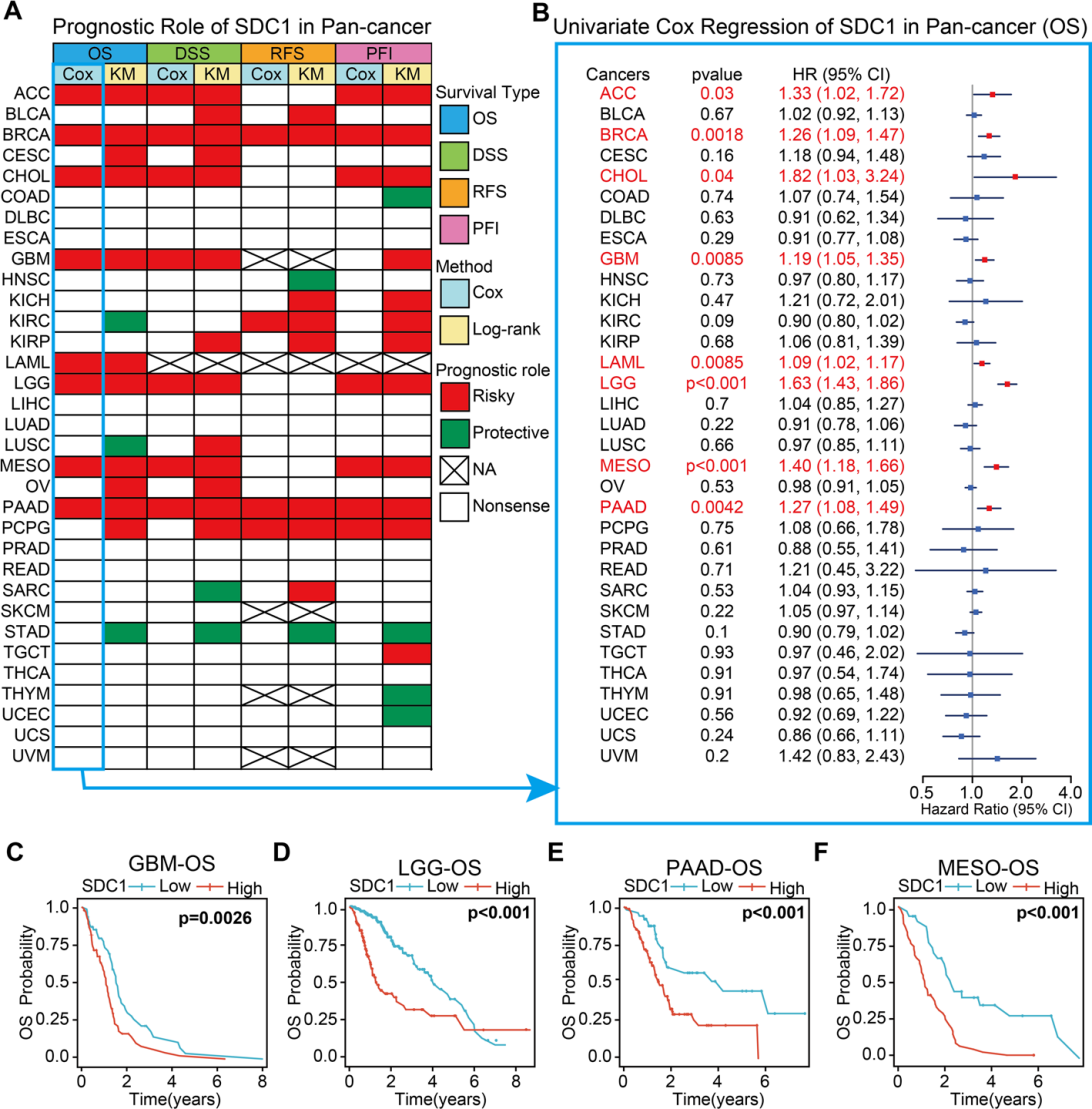
**A** Heatmap illustrating the association of SDC1 expression with four survival outcomes: OS, DSS, RFS, and PFI. The analysis employed log‑rank (Kaplan‑Meier) tests and multivariate Cox regression using clinical outcome data from TCGA. Red tiles indicate risk factors, green tiles denote protective factors; only associations with *P* < 0.05 were shown. **B** Summary of the univariate Cox regression analysis evaluating the prognostic impact of SDC1 across multiple malignancies. Malignancies marked in red highlight those where elevated SDC1 expression demonstrated a statistically significant association with adverse outcomes **C**–**F** OS curves stratified by SDC1 expression in GBM, LGG, PAAD, and MESO

We comprehensively examined SDC1’s prognostic relevance by assessing four survival endpoints—OS, DSS, RFS, and PFS—across multiple cancers. Clustering analysis indicated SDC1 expression correlated with prognosis in 23 cancer types, though not in Lymphoid Neoplasm Diffuse Large B-cell Lymphoma (DLBC), ESCA, Liver hepatocellular carcinoma (LIHC), Prostate adenocarcinoma (PRAD), READ, Lung adenocarcinoma (LUAD), THCA, Skin Cutaneous Melanoma (SKCM), Uterine Carcinosarcoma (UCS), or Uveal Melanoma (UVM) (Fig. 3A). To minimize confounding from non-oncological mortality, DSS was analyzed first. Higher SDC1 levels were associated with poorer DSS in GBM, LGG, Adrenocortical carcinoma (ACC), Bladder Urothelial Carcinoma (BLCA), BRCA, Cervical squamous cell carcinoma and endocervical adenocarcinoma (CESC), Cholangiocarcinoma (CHOL), KIRP, Lung squamous cell carcinoma (LUSC), Mesothelioma (MESO), Ovarian serous cystadenocarcinoma (OV), PAAD, and Pheochromocytoma and Paraganglioma (PCPG), whereas lower levels were protective in SARC and Stomach adenocarcinoma (STAD). RFS and PFI analyses supported these trends. SDC1 consistently showed a protective pattern in STAD, suggesting its potential as a biomarker for gastric cancer progression.

Univariate Cox regression across 33 TCGA cancers further clarified SDC1’s prognostic role. Elevated SDC1 was significantly associated with shorter OS in ACC (HR = 1.33, *P* = 0.03), BRCA (HR = 1.26, *P* = 0.0018), CHOL (HR = 1.82, *P* = 0.04), GBM (HR = 1.19, *P* = 0.0085), Acute Myeloid Leukemia (LAML) (HR = 1.09, *P* = 0.0085), LGG (HR = 1.63, *P* < 0.001), MESO (HR = 1.40, *P* < 0.001), and PAAD (HR = 1.27, *P* = 0.0042) (Fig. 3B). Kaplan–Meier curves confirmed that high SDC1 expression was associated with worse survival in GBM, LGG, PAAD, and MESO (Fig. 3C–F). Independent validation using HPA datasets linked SDC1 to adverse prognosis in BRCA, PAAD, and Kidney renal clear cell carcinoma (KIRC) (Fig. S1E–G).

### GSEA analysis

**Fig. 4:**
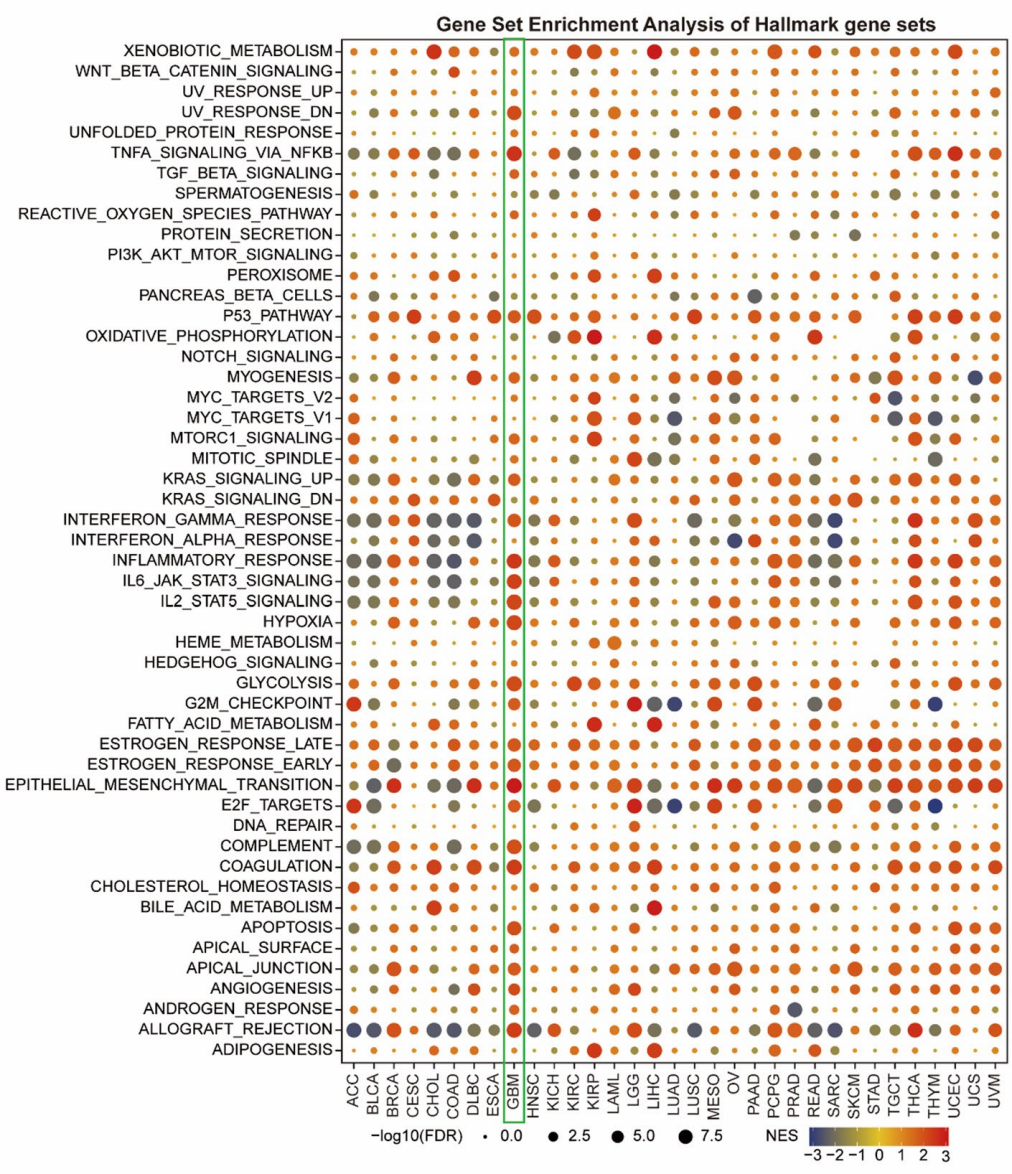
GSEA for SDC1 across cancers. The bubble plot displays GSEA results comparing SDC1-high and SDC1-low tumor patients. Circle dimensions indicate the statistical significance level (P‑value), while the color gradient from red through yellow to blue reflects the corresponding normalized enrichment score (NES) magnitude

GSEA was conducted using DEGs identified between patients stratified into high- and low-SDC1 expression subgroups to clarify SDC1’s functional roles in tumor development. The heatmap illustrates marked enrichment of proliferation-associated pathways—including tumor necrosis factor alpha/nuclear factor kappa B (TNFA/NF-κB) signaling and early and late estrogen response pathways—in SDC1-high tumors. These findings suggest that SDC1 may be involved in hormone-sensitive growth regulation and may function downstream of NF-κB signaling (Fig. 4). Immunological signaling pathways (IFN-α/γ response, inflammatory response, allograft rejection) displayed cancer-specific enrichment. Positive associations were observed in 10 cancer types, including GBM, LGG, BRCA, and CESC, while negative correlations were observed in 9 others, including ACC, BLCA, and CHOL, identifying SDC1 as correlating with gene signatures of immune modulation. Moreover, elevated SDC1 correlated with EMT induction across most cancers, potentially through its interplay with E-cadherin and other adhesion proteins that regulate epithelial integrity and, when dysregulated, foster invasiveness [24]. In high-SDC1 GBM samples, widespread pathway enrichment was observed.

### Expression patterns of SDC1 in various immune and molecular subtypes

Tumor heterogeneity is primarily characterized by immunological and molecular subtyping. Subtypes delineate diverse immune microenvironment characteristics—such as cellular composition, functional states, and immunosuppressive networks—along with variations in oncogenic drivers and tumor cell plasticity. These classification systems critically inform therapeutic decision-making and prognostic evaluation. For instance, in bladder cancer, immune subtypes facilitate the identification of patients potentially suitable for mRNA vaccine therapy targeting MMP9 and IGF2BP2 [25]. In contrast, in breast cancer, molecular subtypes determine the functional roles of immune cells within the tumor microenvironment [26]. Consequently, investigating SDC1 expression patterns across pan-cancer immune and molecular subtypes may elucidate its participation in immunomodulatory processes, with important implications for therapeutic development.

Through the TISIDB portal, we analyzed SDC1 expression disparities across immune and molecular subtypes. Evaluation of immune subtypes revealed statistically significant differences in SDC1 expression across 16 malignancies, including GBM and LGG (Fig. S2 ). Significant expression differences were observed among immune subtypes C1( wound healing), C4 (lymphocyte depletion), and C5 (immunologically quiet) in GBM. While in LGG, variations were observed among subtypes C3 (inflammatory), C4, C5, and C6 (TGF-b dominant).

Analysis of molecular subtypes similarly revealed substantial heterogeneity in SDC1 expression across 15 cancer types (Fig. S3). The expression level of SDC1 was also significantly correlated with GBM and LGG. In GBM patients, Our analysis revealed that SDC1 expression varied significantly among the subtypes, with the Mesenchymal subtype showing the highest expression levels. Furthermore, marked differences in SDC1 abundance were observed among the six molecular subtypes, and the G-CIMP-low subtype of LGG showed the highest level.

### TIMER immune cell infiltration analysis

**Fig. 5:**
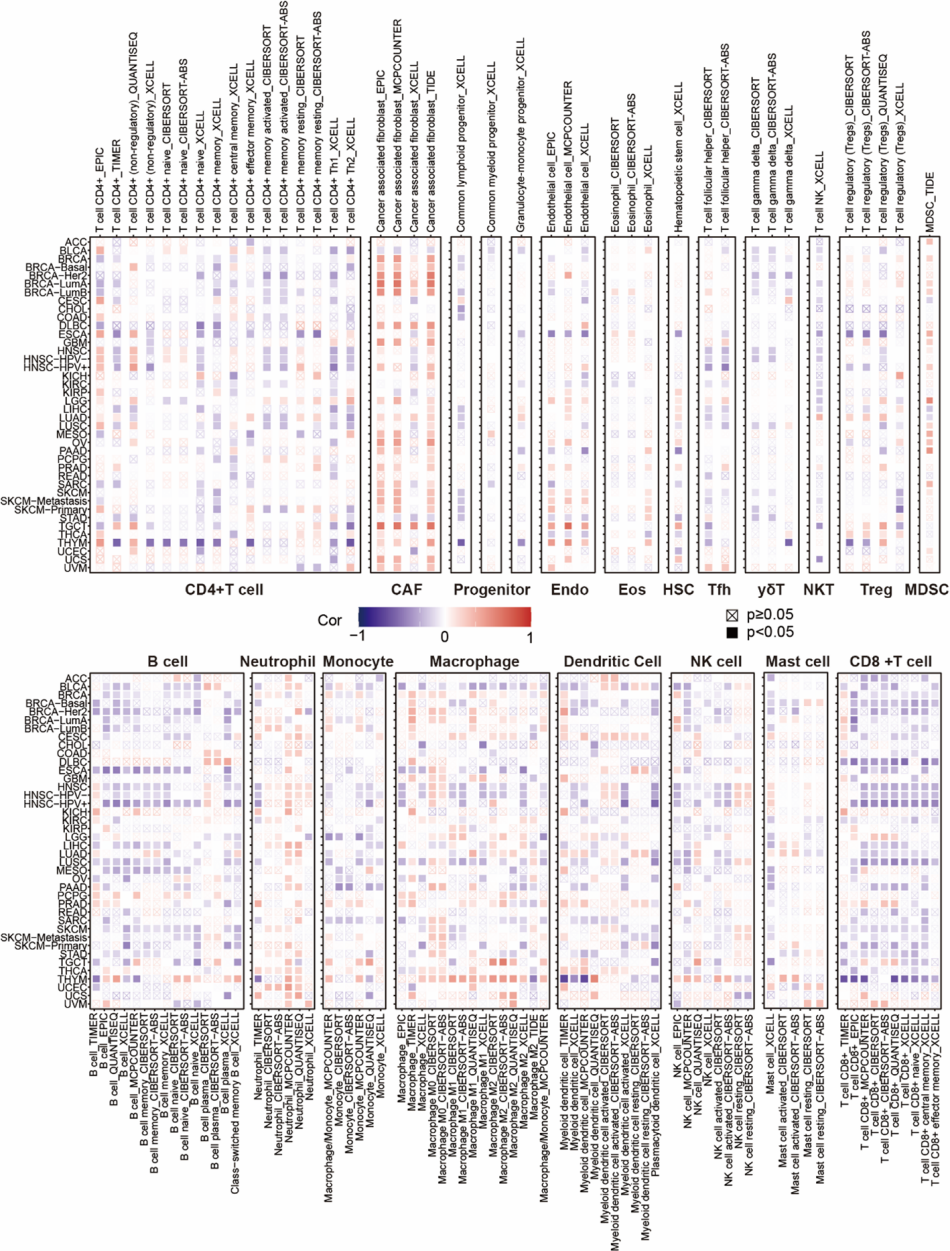
Employing multiple computational algorithms, TIMER2.0 assessed the relationships between SDC1 expression and the abundance of 21 distinct immune cell populations across various human malignancies

The TIMER2.0 platform was utilized to interrogate potential correlations between SDC1 expression levels and the extent of immune cell infiltration. Analysis revealed inverse correlations between SDC1 and multiple lymphocyte populations—CD4 + T cells, lymphoid progenitors, Tfh, γδT, NKT cells, B lymphocytes, monocytes, NK cells, and CD8 + T cells (Fig. 5). These negative associations were especially evident in BRCA, ESCA, HNSC, LUSC, and SKCM. Since CD8 + T lymphocytes mediate tumor cell killing through perforin/granzyme secretion and Fas/FasL pathways [27, 28], and B cells/tertiary lymphoid structures predict immunotherapy efficacy [29], SDC1 may influence T and B cell trafficking, thereby affecting patient prognosis and treatment response.

In contrast, SDC1 expression was positively correlated with immunosuppressive cell populations, including CAFs, MDSCs, and neutrophils, in most cancer types. Significant positive correlations with CAF infiltration were observed in 15 malignancies, including GBM, LGG, BRCA, and DLBC. These pro-tumorigenic populations promote immune evasion and suppress the microenvironment. Together, these observations illuminate SDC1’s role in shaping the immunologic landscape and modulating antitumor immunity across human cancers.

### Drug sensitivity analysis

**Fig. 6:**
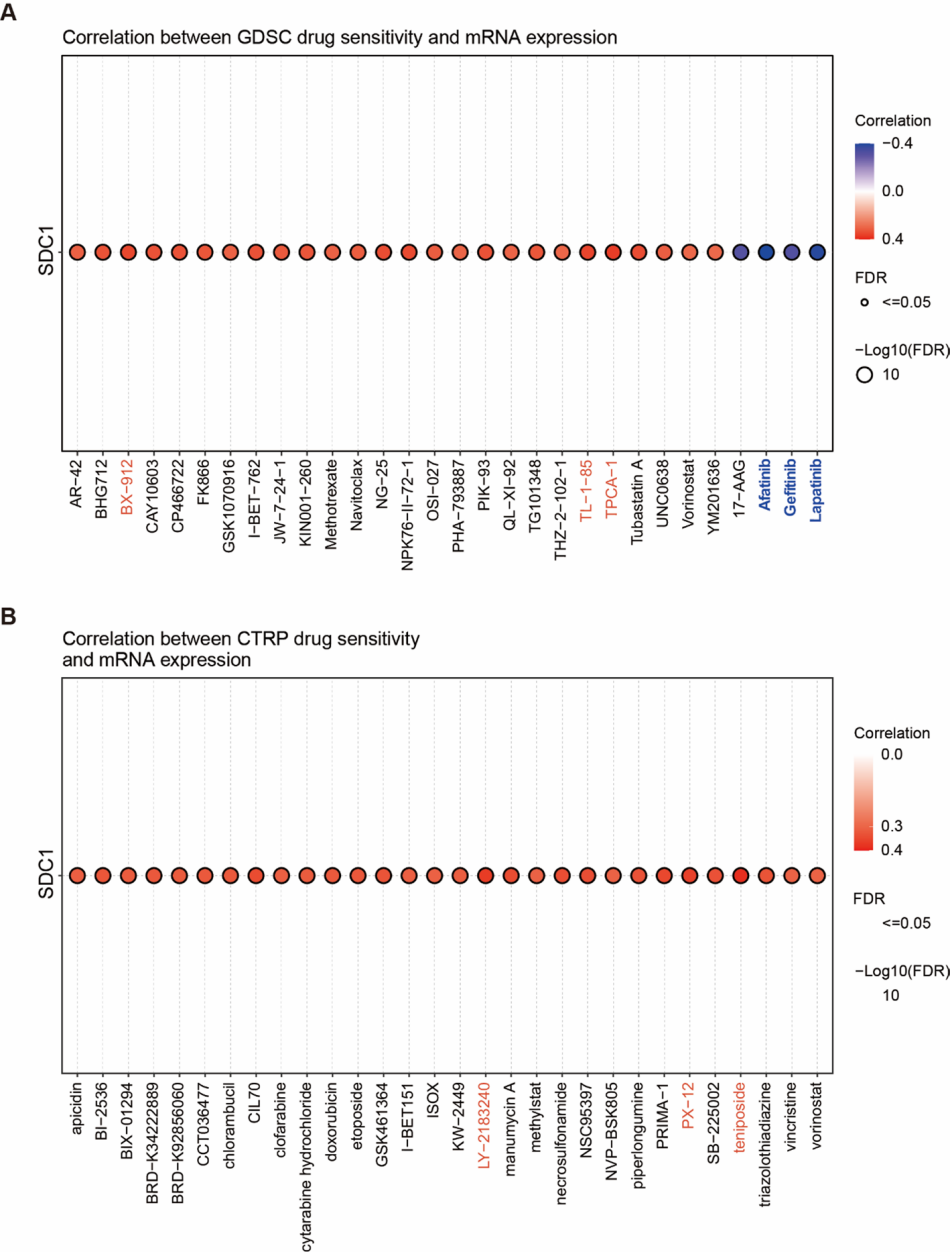
**A**, **B** Association of SDC1 expression with pharmacological response based on GDSC and CTRP datasets

Gene expression signatures serve as valuable indicators of tumor cell responsiveness to conventional chemotherapeutic and molecularly targeted treatments, underscoring their potential to guide therapeutic decisions. By leveraging pharmacogenomic data from the GDSC and CTRP repositories, our analysis revealed a significant correlation between SDC1 expression levels and therapeutic response profiles. Based on the GDSC analysis presented, the compounds exhibiting the strongest positive correlation with SDC1 expression included TPCA-1, TL-1-85, and BX-912 (Fig. 6A). TPCA-1 possesses recognized anti-inflammatory characteristics applicable to both autoimmune pathologies and oncological contexts [30, 31], whereas the mechanistic basis of TL-1-85 remains to be elucidated. BX-912 is a small-molecule PDK1 inhibitor used in cancer target validation studies [32]. In contrast, the most substantial inverse correlations were detected with Afatinib, Lapatinib, and Gefitinib. Gefitinib and Afatinib are representative first- and second-generation inhibitors targeting the EGFR tyrosine kinase, respectively, with principal applications in lung cancer therapeutics [33]. Lapatinib is a dual EGFR/HER2 inhibitor with blood-brain barrier penetration, predominantly used in breast cancer management [34].

Interrogation of the CTRP database depicted identified Teniposide, LY-2,183,240, and PX-12 as displaying the most pronounced positive correlations with SDC1 expression (Fig. 6B). Teniposide finds extensive application in chemotherapeutic investigations for neuroblastoma, lymphoma, and pulmonary malignancies [35–37]. These convergent observations across independent pharmacological platforms support the hypothesis that SDC1 could be considered a transcriptional signature associated with resistance pathways to specific chemotherapeutic and molecularly targeted treatment modalities.

### Clinical correlation and prognostic role of SDC1 in glioma patients

**Fig. 7:**
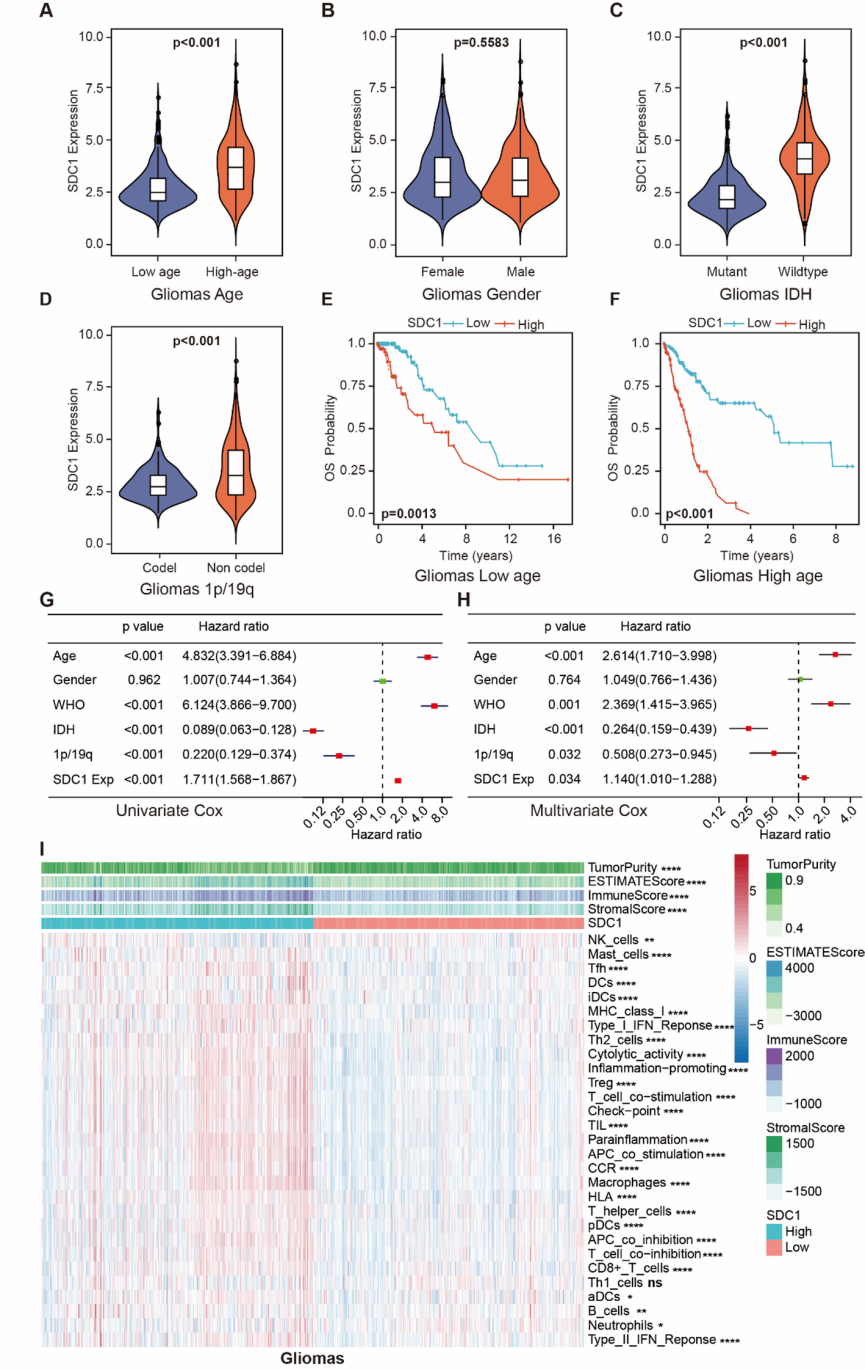
Violin plots showing differences in SDC1 expression across clinical variable subgroups in gliomas. **A** age, **B** gender, **C** IDH, **D** 1p/19q. Assessment of SDC1 prognostic relevance in gliomas. **E** OS survival curve for SDC1 in the glioma low-age subgroup. **F** OS survival curve for SDC1 in the glioma high-age subgroup. **G**–**H** Univariate and multivariate Cox proportional hazards regression models were applied to assess the prognostic impact of SDC1 expression in gliomas. **I** Heatmap illustrating the association between SDC1 transcriptional levels and immune infiltration in gliomas

We examined SDC1 expression patterns in relation to key clinical parameters using data from 143 GBM and 449 LGG patients from TCGA. In the integrated glioma cohort, we observed significant associations between SDC1 expression and patient age, IDH mutation status, and 1p/19q co-deletion status. In contrast, no statistically significant correlation was detected with gender (Fig. 7A-D). Among LGG specimens, increased SDC1 levels were associated with older age and IDH wild-type status, but showed no significant correlation with gender or 1p/19q co-deletion status ( Fig. S4A-D). In GBM cases, SDC1 showed substantial associations with all clinical features except gender, with elevated expression in higher-grade tumors (Fig. S4E-H).

Survival assessment across glioma subgroups indicated that elevated SDC1 expression consistently predicted poorer outcomes in both younger and older patient populations (Fig. 7E, F). Extended survival results for LGG, GBM, and WHO grade-stratified subgroups are provided in Fig. S4I-P.

Prognostic factor analysis through Cox regression modeling incorporated SDC1 expression alongside conventional clinical variables. Univariate Cox proportional-hazards regression revealed significant associations between survival outcomes and multiple clinical and molecular features, namely age, WHO histological grade, IDH mutational profile, 1p/19q codeletion status, and SDC1 expression levels (Fig. 7G). A follow-up multivariate model further validated that higher SDC1 expression independently correlates with reduced OS in glioma [HR = 1.14, 95% CI 1.010–1.288, *p* = 0.034] (Fig. 7H). Therefore, SDC1 is significantly and independently associated with adverse prognosis in glioma.

### ssGSEA analysis

To characterize the immune landscape associated with SDC1 expression, we performed ssGSEA to evaluate 29 immune signatures in glioma samples. Additionally, we calculated immune, stromal, and estimate scores using the ESTIMATE algorithm to assess tumor microenvironment composition [38–41]. We employed ssGSEA to evaluate 29 immune signatures in glioma samples. Analysis of TCGA datasets demonstrated that higher SDC1 levels were positively correlated with increased infiltration of most immune cell types. Enhanced SDC1 levels corresponded with elevated activity in immune functional pathways, including MHC class I presentation, interferon responses, cytolytic function, and T-cell co-stimulation (Fig. 7I).

In LGG cases, SDC1 expression displayed significant associations with 18 immune features, including CD8 + T cells, mast cells, HLA expression, and immune checkpoint markers (Fig. S5A). Among the distinct molecular subtypes of GBM, ssGSEA revealed significant heterogeneity in immune cell infiltration. Notably, the Neural and Mesenchymal subtypes exhibited significantly higher levels of immune cell infiltration compared with the other subtypes (Fig. S5B). These findings suggest that SDC1 may play a role in modulating the immune microenvironment, particularly in GBM molecular subtypes characterized by higher immune infiltration.

Assessment of immune scoring metrics showed that while SDC1 did not significantly correlate with immune scores in GBM or LGG, it maintained positive relationships with other scoring parameters across glioma subgroups (Fig. S4Q-Y). These findings demonstrate distinct immune activation patterns across SDC1 expression subgroups, pointing to its potential utility as a predictive biomarker for therapy response in glioma patients.

### SDC1 enhances GBM cell proliferation, motility, and invasiveness

**Fig. 8:**
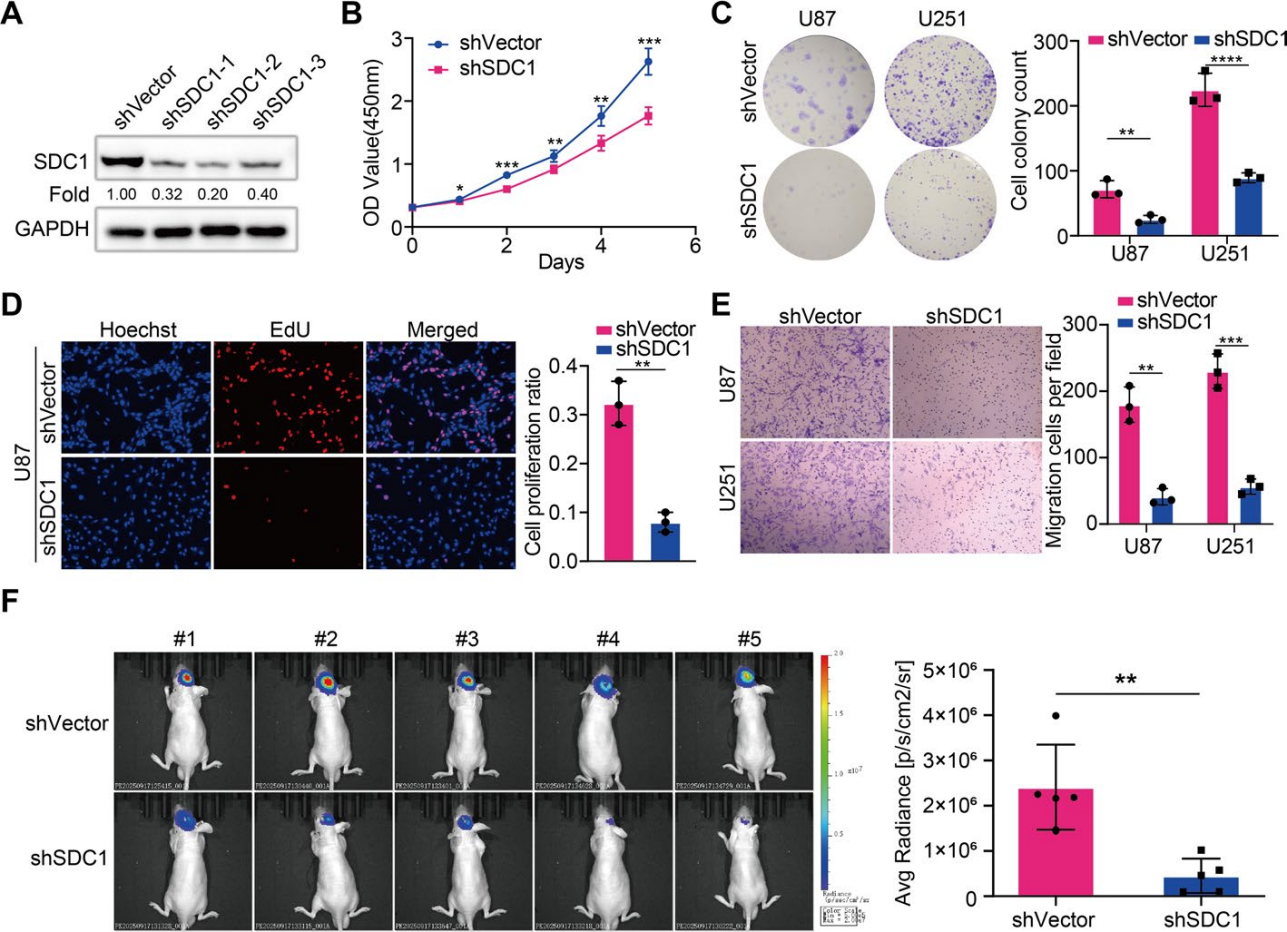
Silencing SDC1 expression suppresses proliferative, migratory, and invasive capacities in U87 and U251 glioblastoma cell lines. **A** Western blot bands show that SDC1 knockdown significantly reduces SDC1 protein abundance in GBM cell models. Fold: fold change. **B** CCK-8 assay measuring optical density values at 450 nm for sh-SDC1 and sh-Vector groups. **C** Colony formation ability was significantly reduced in sh-SDC1 cells. **D** DNA synthesis capacity was decreased in U87 shSDC1 cells. **E** The invasive potential of U87 and U251 glioblastoma cells was markedly attenuated following SDC1 knockdown, as assessed by Transwell invasion assays. **F** Intracranial bioluminescence images and differences in bioluminescence intensity captured from nude mice injected with sh-SDC1 and sh-Vector cells

To functionally characterize SDC1 in GBM, we performed knockdown experiments in selected GBM cell lines, U87 and U251, following expression screening. Western blot analysis validated effective protein downregulation using three distinct shRNA constructs (sh-SDC1-1, sh-SDC1-2, sh-SDC1-3) compared to vector controls (Fig. 8A). Transcript-level suppression was confirmed through qRT-PCR, demonstrating significantly reduced SDC1 mRNA expression in knockdown groups (Fig. S6A).

Proliferation assessment via CCK-8 assays revealed a substantial decrease in viability in SDC1-deficient cells (Fig. 8B). Clonogenic potential was similarly impaired, with both cell lines showing reduced colony formation capacity after SDC1 silencing (Fig. 8C). EdU incorporation assays further confirmed diminished DNA synthesis in SDC1-knockdown U87 cells (Fig. 8D), with consistent results in U251 cells (Fig. S6B), collectively indicating SDC1’s critical role in maintaining proliferative and clonogenic properties.

For migration and invasion evaluation, we conducted Transwell assays without Matrigel and wound healing experiments. SDC1 suppression significantly attenuated both migratory capacity and invasive potential across experimental models (Fig. 8E, Fig. S6C), establishing SDC1’s involvement in GBM cell motility and invasion processes.

### In vivo experiment with SDC1 in nude mice

We assessed SDC1’s role in GBM progression through intracranial xenograft models using luciferase-expressing control and SDC1-knockdown cells. Mice implanted with SDC1-deficient cells showed a substantially decreased tumor burden and delayed growth dynamics compared with controls (Fig. 8F). This in vivo evidence confirms SDC1 promotes tumor expansion and represents a negative prognostic marker in GBM. Combined with our prior findings, these results establish SDC1 as a significant contributor to GBM genesis and disease advancement.

## Discussion

SDC1 is a transmembrane proteoglycan with multifaceted biological roles [42]. Its frequent overexpression in human cancers has been implicated in driving tumor proliferation and promoting epithelial-mesenchymal transition [43], establishing it as a compelling target for understanding cancer progression, prognosis, and treatment resistance [44]. This work represents the first comprehensive pan-cancer investigation of SDC1 using publicly available genomic data, revealing its potential as a prognosis prediction, immune regulator, and targeted therapy.

Assessment of TCGA and GTEx datasets showed SDC1 upregulation in 22 cancer types and downregulation in 5 others, indicating tissue-specific functions. We confirmed elevated SDC1 expression in GBM versus normal brain tissue. SDC1 genomic alterations were associated with poorer outcomes in OS, DSS, and PFI across malignancies.

For diagnostic performance, SDC1 achieved exceptional AUC values of 0.969 in PCPG and 0.965 in UCEC, with additional cancers, including BRCA, PAAD, and gliomas, showing AUCs > 0.7. These findings align with previous reports of SDC1’s diagnostic value in pancreatic cancer [45]and glioma classification [46].

Survival analysis across 33 cancers revealed significant prognostic associations in 23 tumor types. Higher SDC1 expression was associated with worse outcomes in 15 cancers, including ACC, BLCA, GBM, and LGG, but with better prognosis in COAD, HNSC, STAD, THYM, and UCEC, consistent with published data [7].

Pathway analysis connected SDC1 to immune regulation through interferon responses, inflammation, and transplant rejection pathways. Immunosuppressive patterns emerged in nine cancers, including ACC and BLCA, whereas immune-activating features were observed in ten malignancies, including GBM and LGG. SDC1 is also associated with proliferation, oxidative stress, and EMT pathways, supporting its involvement in tumor invasion. Previous studies have demonstrated that SDC1 promotes tumor progression in breast cancer and glioma through activation of the MAPK/ERK and NF-κB signaling pathways [24, 47]. Consistent with these observations, our Gene Set Enrichment Analysis (GSEA) revealed that the NF-κB signaling pathway is significantly enriched in GBM patients with high SDC1 expression. These converging lines of evidence suggest that SDC1 may exert its oncogenic effects in GBM, at least in part, through the MAPK/ERK and NF-κB pathways. We will therefore focus on further investigating key factors within these signaling cascades in our future studies.

SDC1 exhibits variable expression profiles associated with distinct immunological and molecular classifications. Analysis of 16 tumor types revealed distinct SDC1 expression profiles across subtypes, indicating its involvement in diverse immune processes.

Notable subtype-specific patterns emerged across immune classifications. In wound healing, increased SDC1 expression in C1 subtypes may support angiogenesis via VEGF-A/ET-1 regulation and PI3K-Akt/VEGF pathway activation [48]. For IFN-γ-dominant C2 (IFN-g dominant) subtypes, SDC1 appears to suppress IFN-γ-STAT1 signaling, while its loss enhances immunogenicity and T-cell cytotoxicity [49]. In inflammatory C3 contexts, SDC1 may facilitate neutrophil migration by forming chemokine complexes [50]. In lymphocyte-depleted C4 microenvironments, SDC1 might impair T-cell activity via the PD-L1/PD-1 axis or IL-10 secretion [51]. For immunologically quiet C5 subtypes, SDC1’s heparan sulfate chains help distinguish PMN-MDSCs from neutrophils, suggesting that alterations in the SDC1-HS axis are correlated with MDSC activity [52]. In TGF-β-driven C6 subtypes, SDC1 may affect radiotherapy response via TGF-β signaling [53].

Molecular subtype analysis further confirmed SDC1’s differential expression across cancers, supporting its role in tumor-specific pathways. Immune profiling showed SDC1 inversely correlates with antitumor lymphocytes (CD8 + T, CD4 + T, NK cells) but positively associates with immunosuppressive elements (CAFs, MDSCs), consistent with findings that SDC1 blockade improves anti-PD-1 efficacy [49].Notably, the consistency between SDC1-associated immune cell abundance (assessed by TIMER, CIBERSORT, xCell) and immune functional signatures (assessed by ssGSEA) in GBM further supports its potential role in modulating anti-tumor immunity within this malignancy.To assess the clinical relevance of SDC1, we examined its association with pharmacological responses. EGFR promotes cancer cell proliferation and division by phosphorylating downstream proteins and activating signaling pathways [54]. Pharmacogenomic assessment linked elevated SDC1 with enhanced sensitivity to EGFR inhibitors Afatinib, Lapatinib, and Gefitinib. Given SDC1’s coreceptor function in amplifying EGFR signaling via HS chains [55], our correlative findings suggest that SDC1 expression is linked to TKI efficacy, supporting its potential utility as a predictive biomarker for TKI therapy.The WHO classifies gliomas into four histological grades. LGG (WHO II-III) shows differentiated features but may progress. At the same time, GBM, classified as WHO grade IV, constitutes the most malignant primary brain tumor in adults, with a five-year survival rate as low as 7.2% [56]. Current treatments combining surgery, radiation, and temozolomide often fail due to local recurrence [57], highlighting the need for new therapeutic targets.

We examined SDC1’s role in glioma, given its pan-cancer importance. Patients with IDH-mutant tumors and 1p/19q co-deletion typically have a more favorable prognosis [58, 59]. Our analysis indicated that elevated SDC1 levels were associated with IDH wild-type and 1p/19q non-codeletion in tumors. TCGA analysis linked elevated SDC1 to reduced survival, and Cox models identified it as an independent risk factor. SDC1 also correlated positively with immune, stromal, and estimate scores, indicating involvement of the immune microenvironment.

Functional validation in GBM models showed SDC1 knockdown suppressed proliferation, invasion, migration, and tumor growth. Our multi-platform analysis establishes SDC1’s oncogenic role and supports its potential as a prognostic biomarker and immunotherapeutic target.

Several limitations should be acknowledged in the present study. First, the sample sizes across cancer types in public databases are uneven, and potential methodological variability between data sources may exist. Second, while we demonstrated SDC1’s prognostic value, its precise immunomodulatory mechanisms remain to be investigated. Additional in vitro and in vivo studies are needed to fully elucidate SDC1’s oncogenic functions and validate its clinical utility. Third, it should be noted that multivariate Cox regression analysis with adjustment for critical clinical covariates was performed exclusively in the glioma cohort. In contrast, the prognostic findings across various cancer types were derived from univariate analyses alone. Consequently, the independent prognostic relevance of SDC1 in other malignancies—such as BRCA and PAAD—remains to be firmly established. These findings thus warrant cautious interpretation and necessitate further validation through multivariate adjustment in future investigations.

## Conclusion

SDC1 emerges as a candidate biomarker with pan-cancer applicability, especially in GBM. Its connections with immune infiltration further support exploring SDC1 as a target for immunotherapy development.

## Electronic Supplementary Material

Below is the link to the electronic supplementary material.

Supplementary Material 1.

## Data Availability

All datasets used or analyzed in this study are accessible through publicly available databases. These data can be accessed by logging into the relevant databases. The specific databases and their links are as follows: UCSC Xena (https://xenabrowser.net/datapages/), UALCAN (https://ualcan.path.uab.edu/cgi-bin/ualcan-res.pl), GEPIA3 (https://gepia3.bioinfoliu.com/find\_drug/), cBioPortal (https://www.cbioportal.org/results/cancerTypesSummary? case\_set\_id=all&gene\_list=SDC1&cancer\_study\_list=5c8a7d55e4b046111fee2296), HPA (https://www.proteinatlas.org/ENSG00000115884-SDC1/subcellular), TIMER2 ( [http://timer.cistrome.org/](http:/timer.cistrome.org) ), STRING ( [https://string-db.org/cgi/network? taskId=b2GKHDjEKoD2&sessionId=b8Pcz8hUNnHn /](https:/string-db.org) ), CPTAC ( [https://proteomics.cancer.gov/programs/cptac](https:/proteomics.cancer.gov/programs/cptac) ), TISIDB ( [http://cis.hku.hk/TISIDB/](http:/cis.hku.hk/TISIDB) ), GSCA (https://guolab.wchscu.cn/GSCA/#/).

## Abbreviations

ACC: Adrenocortical carcinoma
ATCC: American Type Culture Collection
AUC: Area under the curve
BLCA: Bladder Urothelial Carcinoma
BRCA: Breast invasive carcinoma
C1: Wound healing
C2: IFN-g dominant
C3: Inflammatory
C4: Lymphocyte depletion
C5: Immunologically quiet
C6: TGF-b dominant
CAFs: Cancer-associated fibroblasts
CESC: Cervical squamous cell carcinoma and endocervical adenocarcinoma
CHOL: Cholangiocarcinoma
CI: Confidence intervals
COAD: Colon adenocarcinoma
CPTAC: Clinical Proteomic Tumor Analysis Consortium
DEGs: Differentially expressed genes
DLBC: Lymphoid Neoplasm Diffuse Large B-cell Lymphoma
DMEM: Dulbecco’s Modified Eagle Medium
DSS: Disease-specific survival
ESCA: Esophageal carcinoma
FBS: Fetal bovine serum (Gibco)
FDR: False Discovery Rate
GBM: Glioblastoma
GSEA: Gene Set Enrichment Analysis
GSVA: Gene Set Variation Analysis
GTEx: Genotype-Tissue Expression
HNSC: Head and Neck squamous cell carcinoma
HPA: Human Protein Atlas
HR: Hazard ratios
HRP: Horseradish peroxidase
HSCs: Hematopoietic stem cells
KICH: Kidney Chromophobe
KIRC: Kidney renal clear cell carcinoma
KIRP: Kidney renal papillary cell carcinoma
LAML: Acute Myeloid Leukemia
LGG: Lower Grade Glioma
LIHC: Liver hepatocellular carcinoma
logFC: Log fold change
LUAD: Lung adenocarcinoma
LUSC: Lung squamous cell carcinoma
MDSCs: Myeloid-derived suppressor cells
MESO: Mesothelioma
MSigDB: Molecular Signatures Database
NES: Normalized Enrichment Scores
NK: Natural killer
OS: Overall survival
OV: Ovarian serous cystadenocarcinoma
PAAD: Pancreatic adenocarcinoma
PCPG: Pheochromocytoma and Paraganglioma
PFI: Progression-free interval
PPI: Protein-protein interaction
PRAD: Prostate adenocarcinoma
READ: Rectum adenocarcinoma
RFS: Recurrence-free survival
ROC: Receiver operating characteristic
SARC: Sarcoma
SDC1: Syndecan-1
SKCM: Skin Cutaneous Melanoma
ssGSEA: Single-sample Gene Set Enrichment Analysis
STAD: Stomach adenocarcinoma
TCGA: The Cancer Genome Atlas
TGCT: Testicular Germ Cell Tumors
THCA: Thyroid carcinoma
THYM: Thymoma
Tregs: Regulatory T cells
UCEC: Uterine Corpus Endometrial Carcinoma
UCS: Uterine Carcinosarcoma
UVM: Uveal Melanoma
