# Supplementary material for: Integrated pan-cancer analysis and experimental validation reveal that syndecan-1 drives glioblastoma pathogenesis and associates with immune infiltration
Source: Discov Oncol. 2026 May 7;17:955. doi: 10.1007/s12672-026-05172-0 (PMC13319849; doi:10.1007/s12672-026-05172-0)
Supplement: Supplementary file 1 — Supplementary Material 1. [file 12672_2026_5172_MOESM1_ESM.docx]

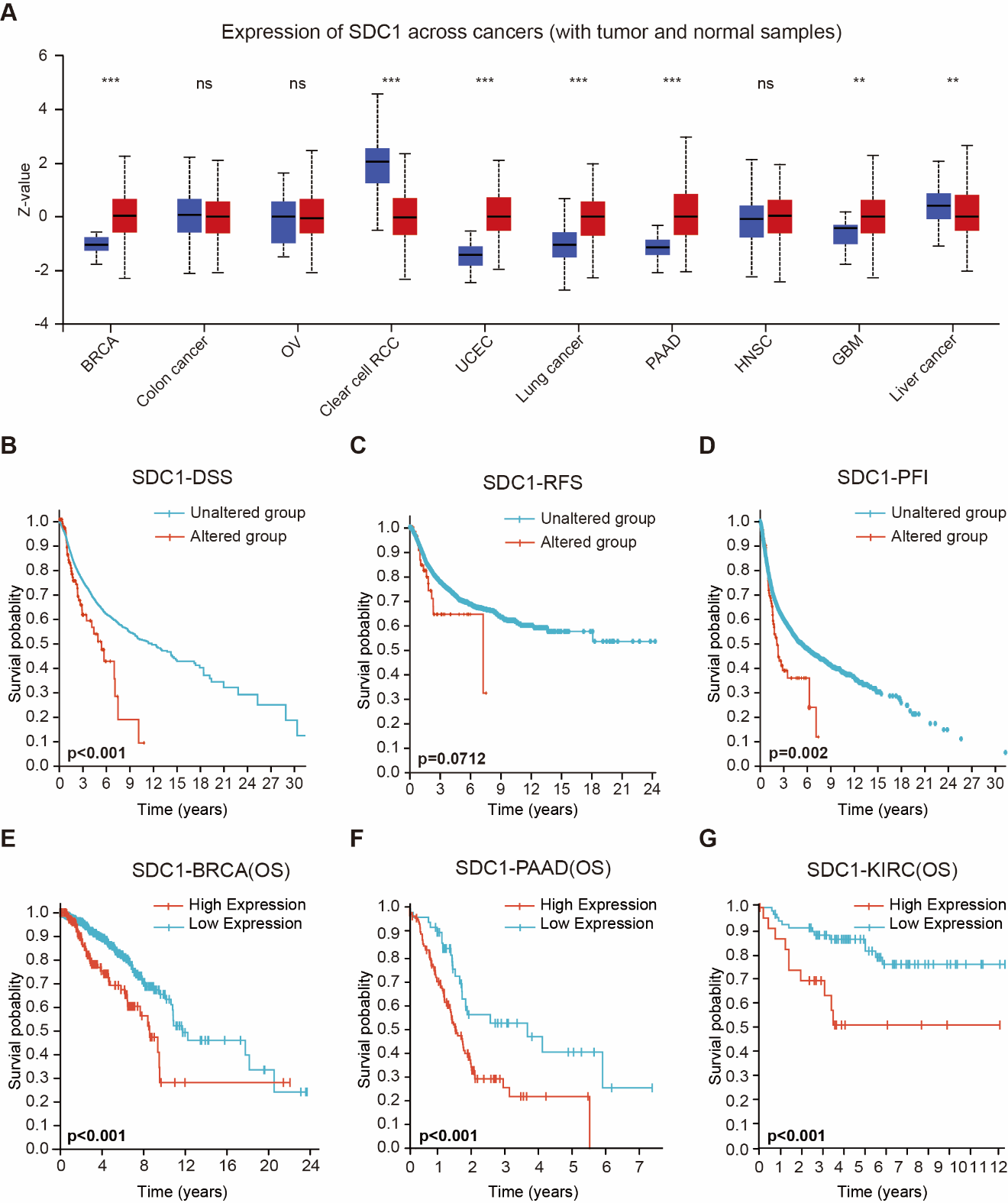


Supplementary Fig. 1: (A) SDC1 protein expression levels between cancer and normal tissues. (B-D) Kaplan-Meier survival curves for SDC1-mutated versus SDC1-non-mutated patients. (B)DSS, (C) RFS, (D) PFI. (E-G) Kaplan-Meier survival curves from the HPA database showing the correlation of SDC1 levels with clinical outcomes across various malignancies. (E) BRCA, (F) PAAD, (G) KIRC


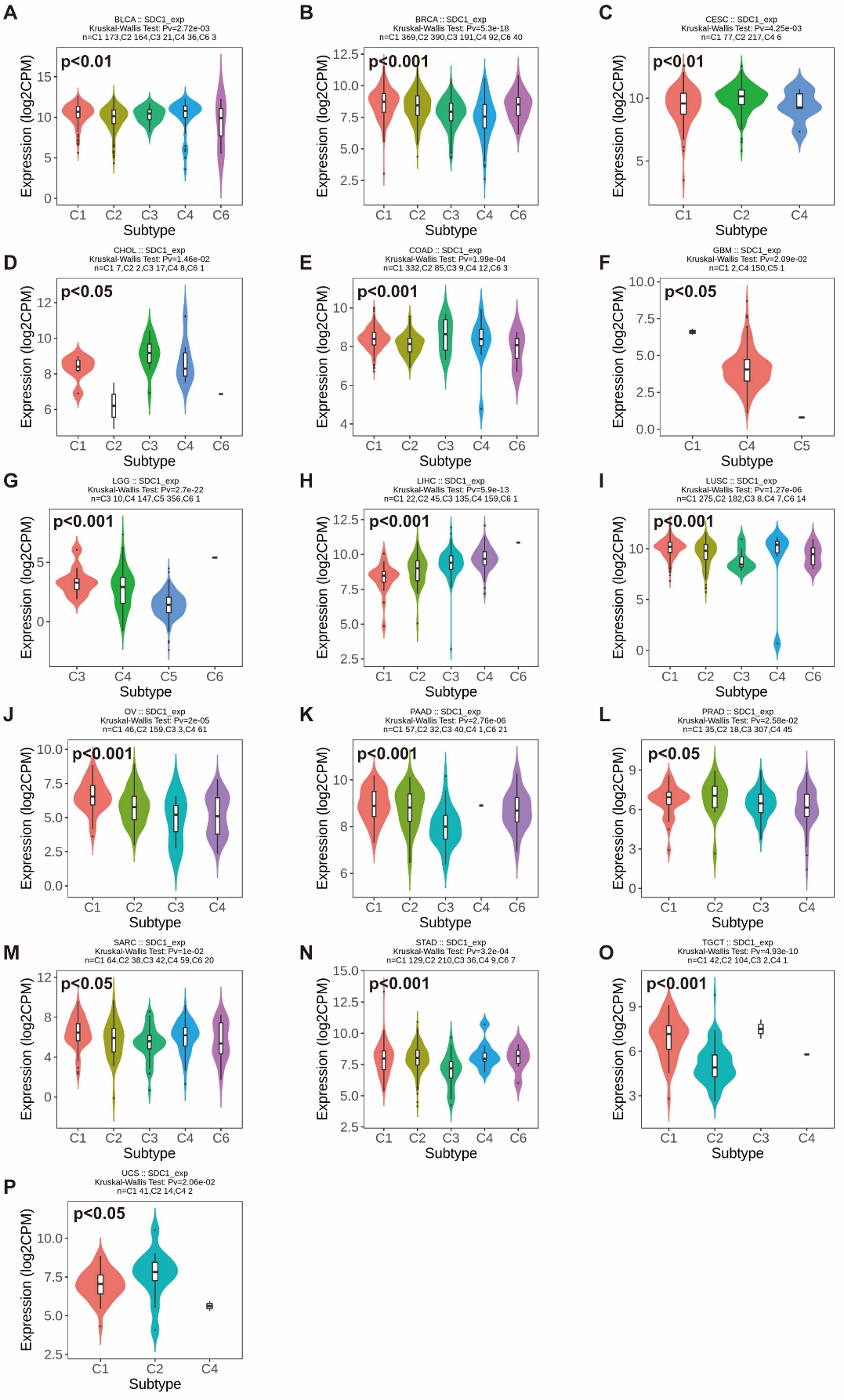


Supplementary Fig. 2: Association between SDC1 expression profiles and immune subtype classification across 16 malignancies.


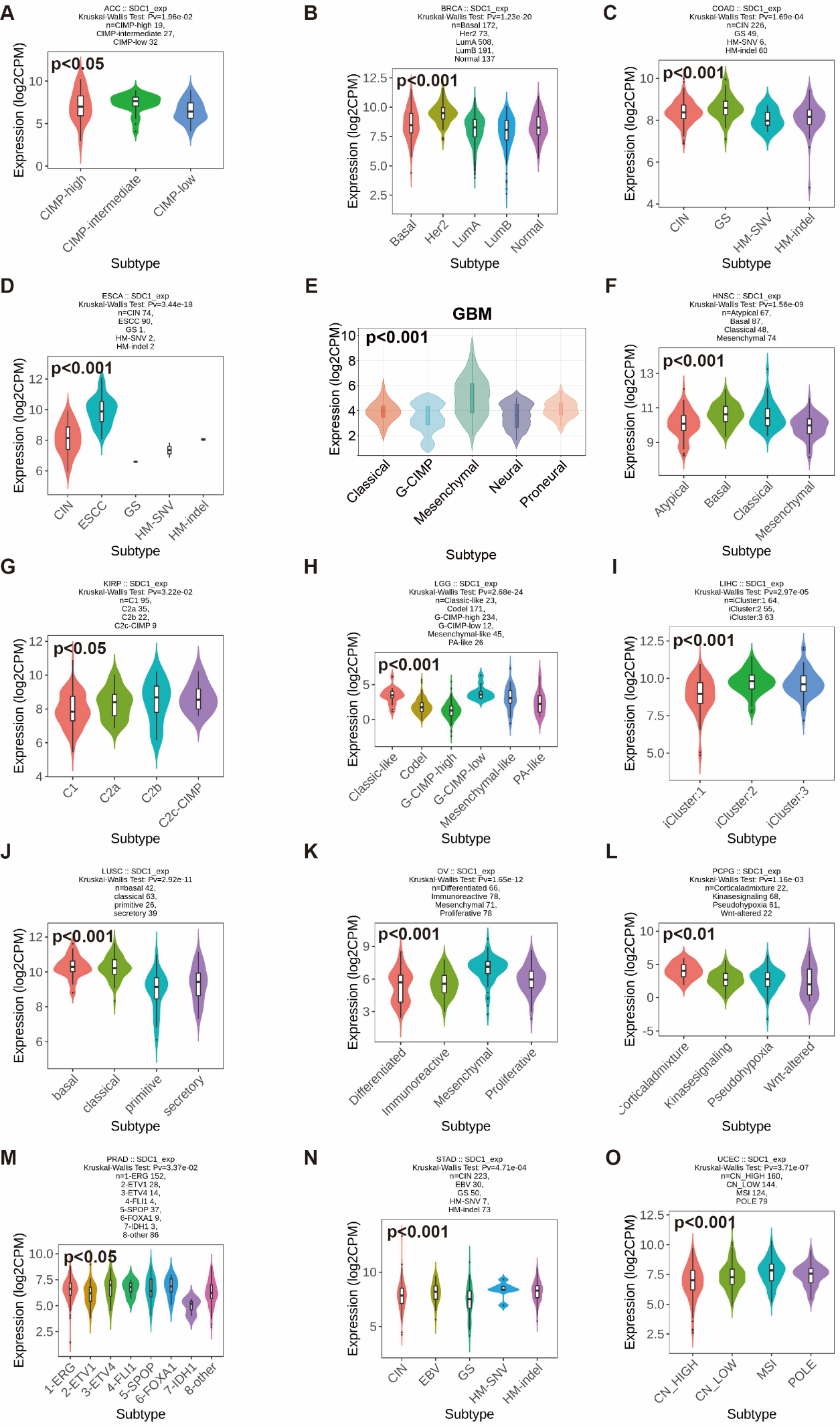


Supplementary Fig. 3: Relationship between SDC1 expression levels and molecular subtype classification in 15 malignancies.


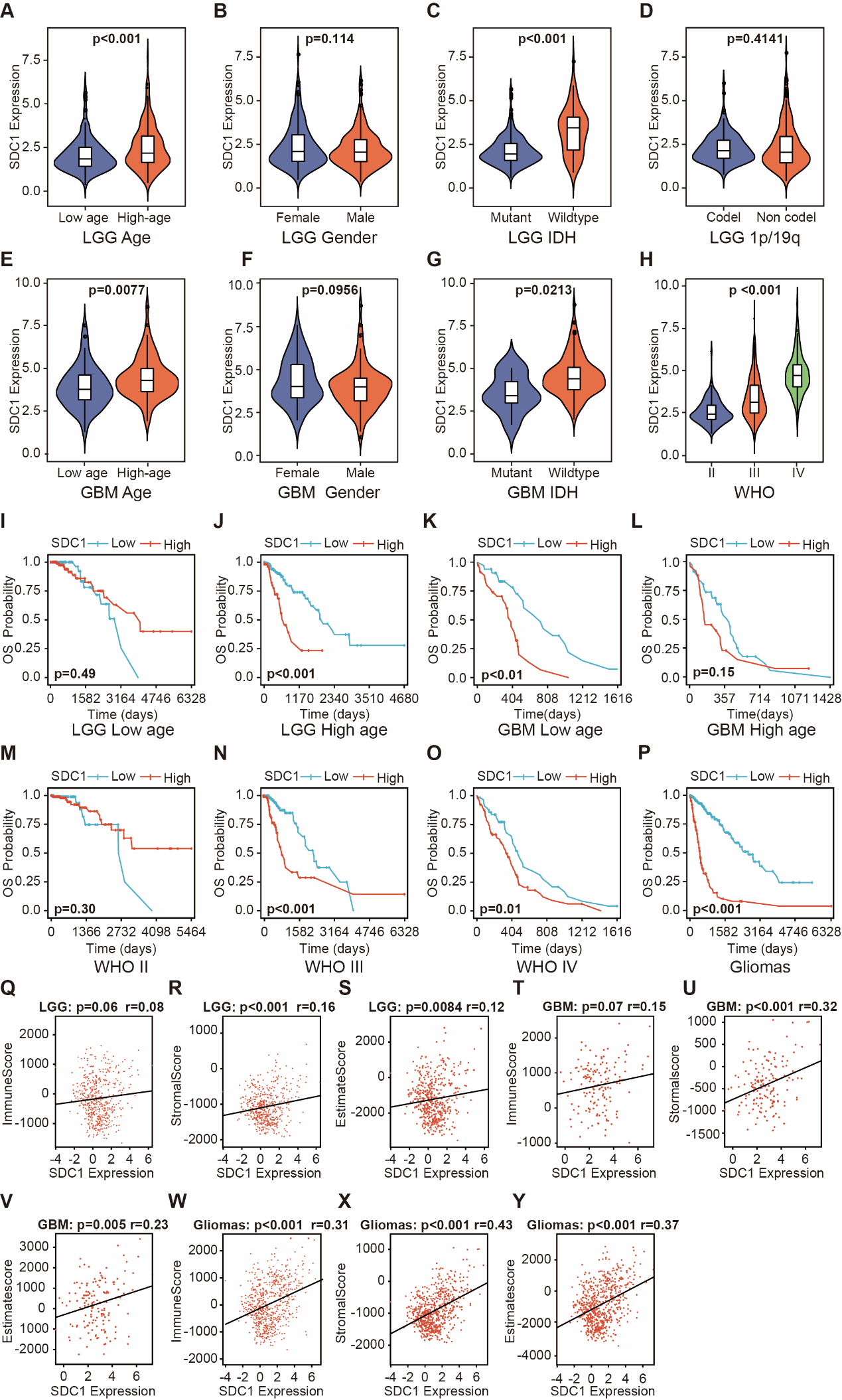


Supplementary Fig. 4: (A-D) Violin plots showing differences in SDC1 expression across clinical variable subgroups in LGG: (A) age, (B) gender, (C) IDH, (D) 1p/19q. (E-H) Violin plots showing differences in SDC1 expression across clinical variable subgroups in GBM: (E) Age, (F) Gender, (G) IDH status, (H) WHO grade. (I-P) Kaplan-Meier survival curves for SDC1 expression in clinical subgroups: (I) LGG low age, (J) LGG high age, (K) GBM low age, (L) GBM high age, (M) WHO II, (N) WHO III, (O) WHO IV, (P) Combined gliomas. (Q-Y) Association analysis between SDC1 expression and immune-related scores in LGG, GBM, and combined gliomas: (Q) LGG immune Score, (R) LGG stromal score, (S) LGG estimate score, (T) GBM immune score, (U) GBM stromal score, (V) GBM estimate score, (W) gliomas immune Score, (X) gliomas stromal Score, (Y) gliomas estimate score.


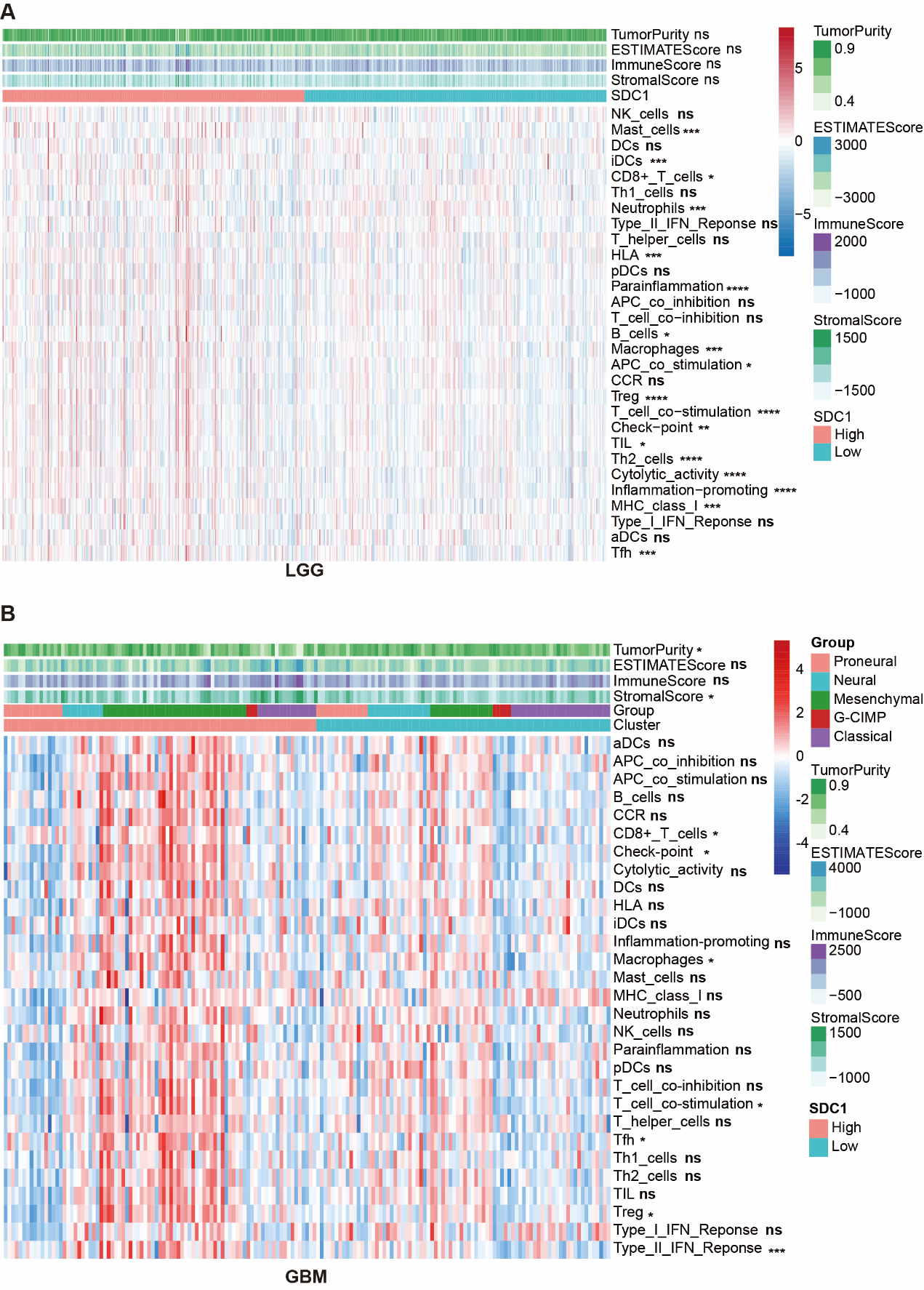


Supplementary Fig. 5: Heatmaps showing the association between SDC1 expression and immune infiltration patterns encompassing both LGG (A) and GBM (B) subtypes.


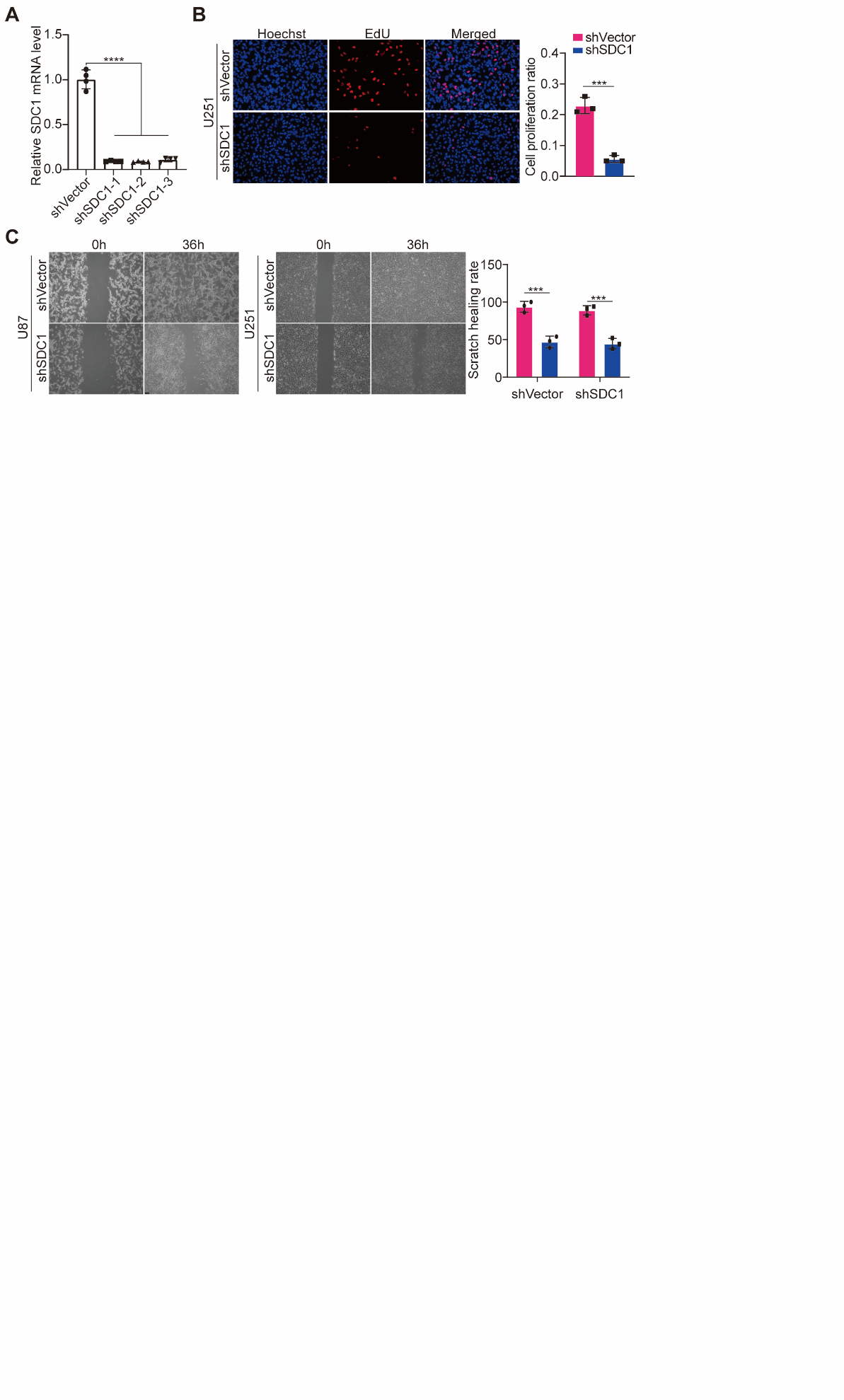


Supplementary Fig. 6: (A) RT-qPCR analysis confirmed a significant reduction of SDC1 mRNA expression in sh-SDC1 cells. (B) DNA synthesis capacity was decreased in U251 shSDC1 cells. (C) Wound closure assays demonstrated that silencing SDC1 expression significantly reduced the migratory potential of both U87 and U251 GBM cell lines.


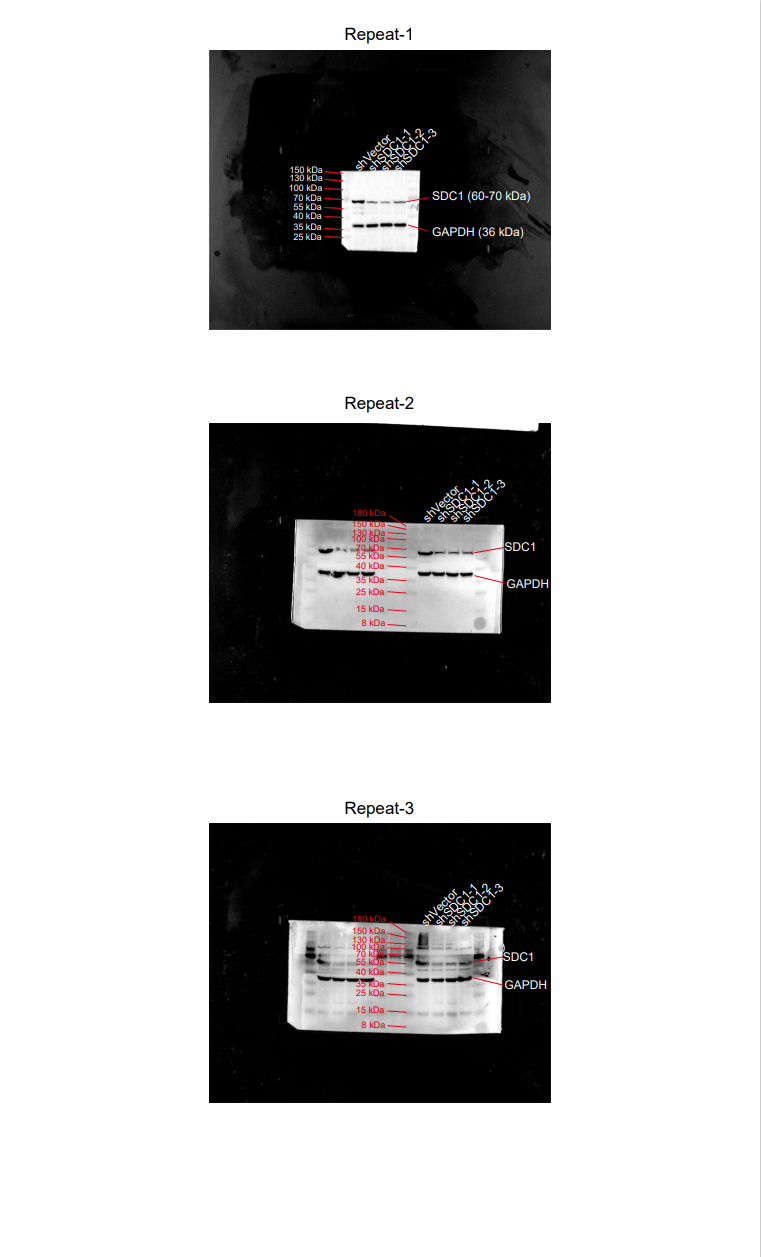


Supplementary Wb Raw Data: full-length, uncropped membrane images with clear edges.
